# Two Cases of Hernia Congenita

**Published:** 1800

**Authors:** Henry Fryer

**Affiliations:** Surgeon at Stamford.


IX. *Two Cafes of Hernia congenita. By Mr.* Henry Fryer, *Surgeon at Stamford. Communicated in a Letter to* John Clarke, *M.D. Phyfician in London; and by him to Dr.* Simmons.

CASE I.

IN 1790 I was confulted by a perfon, neither of whofe tefticles had come down into the fcrotum; by this, I mean, that they had not paffed the abdominal rings, but formed two fmall tumors refembling bubonocele. He informed me that he had been frequently near dying from ftrangulation of the inteftines at both openings, fometimes at one, fometimes the other; inteftine and tefticle being both to be felt at each.

The opinion which he wanted from me was, whether I would advife a trufs to be worn, which had been forbidden by another furgeon, left the tefticles fhould be injured by it. I had no doubt in advifing that he fhould immediately have a double trufs, as from the fize of the openings there was no profpect of the tefticles ever paffing out (an idea which had been held out to him) he being now nearly thirty years old, and particularly as the tumors were both

 readily readily returnable. He followed my advice, and had no trouble from the complaint afterward.

### CASE II.

About three months ſince I was obliged to put on a truſs for a child of Mr. L——'s, of this place, for a ſimilar complaint, with the difference of its being on the *right* ſide only. He had ſuffered, at times, violent pain, on which account I had been conſulted ſome months before; but as the child was only ſix years old, I deſired the parents to wait ſome time, hoping that the teſticle would have made its way down, but in this I was deceived; the ſymptoms of ſtrangulation returned more frequently and with greater violence; I, therefore, thought it would be improper to defer the application of the truſs any longer. Since that time he has remained perfectly well.

The practical concluſion from theſe two caſes is, that hernia inguinalis may exiſt, although the teſticles may not have deſcended into the ſcrotum; and if it ſhould, that the ſame means are both neceſſary and practicable for its relief.

